# Long-term mortality and technique survival in peritoneal dialysis patients: a 25-year retrospective analysis in a single center

**DOI:** 10.1093/ckj/sfaf215

**Published:** 2025-07-08

**Authors:** Roberta Scarmignan, Gaetano Alfano, Niccolò Morisi, Francesco Fontana, Giacomo Mori, Marco Ferrarini, Camilla Ferri, Laura Tonelli, Giulia Ligabue, Riccardo Magistroni, Mariacristina Gregorini, Gabriele Donati

**Affiliations:** Surgical, Medical and Dental Department of Morphological Sciences related to Transplant, Oncology and Regenerative Medicine (CHIMOMO), Section of Nephrology, University of Modena and Reggio Emilia, Modena, Italy; Nephrology, Dialysis and Kidney Transplant Unit, Azienda Ospedaliero Universitaria di Modena, Modena, Italy; Surgical, Medical and Dental Department of Morphological Sciences related to Transplant, Oncology and Regenerative Medicine (CHIMOMO), Section of Nephrology, University of Modena and Reggio Emilia, Modena, Italy; Nephrology, Dialysis and Kidney Transplant Unit, Azienda Ospedaliero Universitaria di Modena, Modena, Italy; Nephrology, Dialysis and Kidney Transplant Unit, Azienda Ospedaliero Universitaria di Modena, Modena, Italy; Surgical, Medical and Dental Department of Morphological Sciences related to Transplant, Oncology and Regenerative Medicine (CHIMOMO), Section of Nephrology, University of Modena and Reggio Emilia, Modena, Italy; Surgical, Medical and Dental Department of Morphological Sciences related to Transplant, Oncology and Regenerative Medicine (CHIMOMO), Section of Nephrology, University of Modena and Reggio Emilia, Modena, Italy; Surgical, Medical and Dental Department of Morphological Sciences related to Transplant, Oncology and Regenerative Medicine (CHIMOMO), Section of Nephrology, University of Modena and Reggio Emilia, Modena, Italy; Surgical, Medical and Dental Department of Morphological Sciences related to Transplant, Oncology and Regenerative Medicine (CHIMOMO), Section of Nephrology, University of Modena and Reggio Emilia, Modena, Italy; Surgical, Medical and Dental Department of Morphological Sciences related to Transplant, Oncology and Regenerative Medicine (CHIMOMO), Section of Nephrology, University of Modena and Reggio Emilia, Modena, Italy; Nephrology, Dialysis and Kidney Transplant Unit, Azienda Ospedaliero Universitaria di Modena, Modena, Italy; Nephrology and Dialysis Unit, AUSL-IRCCS, Arcispedale S. Maria Nuova Reggio Emilia, Reggio Emilia, Italy; Surgical, Medical and Dental Department of Morphological Sciences related to Transplant, Oncology and Regenerative Medicine (CHIMOMO), Section of Nephrology, University of Modena and Reggio Emilia, Modena, Italy; Nephrology, Dialysis and Kidney Transplant Unit, Azienda Ospedaliero Universitaria di Modena, Modena, Italy

**Keywords:** mortality rate, peritoneal dialysis, technique failure, transition to hemodialysis

## Abstract

**Background:**

We comprehensively assessed patient survival and the duration of peritoneal dialysis (PD) treatment over a 25-year period within our PD unit.

**Methods:**

We retrospectively evaluated 497 PD patients who initiated PD between 1996 and 2021. The cohort was divided into three distinct periods based on pivotal events, such as the introduction of more biocompatible dialysis solutions and the initiation of remote monitoring technologies. Kaplan–Meier survival assessments, Cox proportional hazards model and Gray subdistribution hazard model were employed to evaluate patient survival and PD-to-hemodialysis (HD) transfer.

**Results:**

The use of PD as the initial treatment increased significantly over the years. Mean age was 63.5 ± 15.7 years; 61% were male, and 61% had three or more comorbidities including hypertension (81%), dyslipidemia (66%), cardiovascular disease (56%) and diabetes (16%). The five-year mortality rate was 40%. Risk factors for mortality included continuous ambulatory peritoneal dialysis (CAPD) [hazard ratio (HR) = 2.63, 95% confidence interval (CI) 1.76–3.93; *P* < .001], older age (HR = 2.96, 95% CI 1.98–4.43; *P* < .001), cardiovascular disease (HR = 1.96, 95% CI 1.31–2.95; *P* = .001) and the use of renin–angiotensin–aldosterone system inhibitors (RAASi) (HR = 1.81, 95% CI 1.22–2.70; *P* = .004). At 5 years, 48% of patients remained on PD. In the Cox model, risk factors for PD-to-HD transfer included CAPD (HR = 1.62, 95% CI 1.21–2.16; *P* = .001). RAASi use (HR = 0.66, 95% CI 0.46–0.94; *P* = .02) and female sex (HR = 0.70, 95% CI 0.51–0.96; *P* = .03) were associated with longer PD duration.

**Conclusions:**

The study provides insights into the changing landscape of PD. Advances in PD solutions and remote monitoring have contributed to changes in PD outcomes and its increased adoption over the years. Given the observational nature of the study, caution is warranted in interpreting the association of both CAPD and RAASi with mortality.

## Graphical Abstract

**Graphical Abstract:**
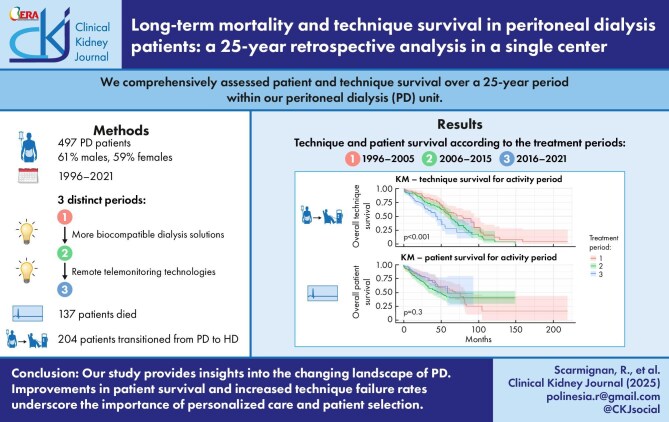

KEY LEARNING POINTS **What was known:**

- Few studies have undertaken comprehensive long-term assessments of patient and technique survival, especially over an extended period, within the peritoneal dialysis (PD) patient population.

**This study adds:**

- This study draws on a quarter-century of real-world data from our PD unit, exploring patient and technique survival, while offering a detailed understanding of the evolving landscape of a PD outpatient clinic.

**Potential impact:**

- Improvements in patient survival and increased technique failure rates underscore the importance of personalized care and patient selection.
- The use of two different statistical methods to assess both patient and technique survival provides insights into the complexity of PD population.

## INTRODUCTION

Over the past few decades, peritoneal dialysis (PD) has emerged as a favorable modality for the management of end-stage kidney disease (ESKD), providing patients with a viable alternative to hemodialysis (HD) [1].

Nevertheless, the success of many PD programs is still hindered by the fact that PD patients continue to face a substantial risk of transitioning from PD to HD due to complications like catheter malfunction, peritonitis and ultrafiltration failure [2, 3].

Current PD research is predominantly focused on optimizing the dialysis process, enhancing patient comfort and improving quality of life [4, 5]. Few studies have undertaken comprehensive long-term assessments of patient and technique survival, especially over an extended period, within the PD patient population [6].

Furthermore, there is a lack of studies that consider the evolution of clinical practices over time in relation to pivotal events in PD management, such as the introduction of more biocompatible or glucose-sparing PD solutions and the implementation of remote monitoring technologies.

Our study addresses this gap by exploring these factors and their potential impact on long-term outcomes. Drawing on a quarter-century of real-world data from our PD unit, we provide a comprehensive analysis of patient survival, technique failure and the factors influencing these outcomes.

## MATERIALS AND METHODS

This is a single-center retrospective study conducted at the Nephrology Dialysis and Kidney Transplant Unit of the Azienda Ospedaliera Universitaria di Modena (Italy). This study uses databases which only contain de-identified data. This study was approved by the Institutional Review Board review, CE AVEN 507/2021/OSS “CKD-Project.” We enrolled all patients who initiated PD in our unit between 1 January 1996 and 31 December 2021. Individuals under 18 years of age were excluded. Informed consent was obtained from individual participants whenever possible. Comparative investigations were carried out among three distinct periods: 1996–2005, 2006–15 and 2016–21. These three periods have been chosen in relation to two substantial changes in our clinical practice: the use of more biocompatible dialysis solutions in the second period and the introduction of remote telemonitoring technology starting in 2016.

### Data collection

The information gathered from the medical records included the age at which PD was initiated, gender, underlying causes of ESKD, the presence of diabetes mellitus (DM), use of renin–angiotensin–aldosterone system inhibitors (RAASi), estimated glomerular filtration rate (eGFR) at the start of PD, the patients’ clinical status before commencing PD, the mean duration of PD utilization, the chosen PD modality, the type of peritoneal catheter used (single- and double-cuff Vicenza catheter, self-locating catheter, straight or coiled swan neck catheter), and the ultimate status of patients at the conclusion of the follow-up period (including whether they had deceased, received kidney transplantation, transitioned to HD or continued with the PD technique). The causes of ESKD were grouped in the following categories: DM, hypertension, vascular disease (including renal artery stenosis, atheroembolic disease and renal vein thrombosis), primary and secondary glomerular disease, tubulointerstitial disease, urinary tract dysfunction/obstruction and other causes.

The following comorbidities were considered: hypertension (defined as the use of antihypertensive drugs), hyperuricemia, hypercholesterolemia and DM. The grade of comorbidity was derived directly from the number of comorbidities: grade 0 (low risk) is a zero score, grade 1 (medium risk) is a score of 1–2, and grade 2 (high risk) a cumulative score of ≥3. Since only five patients from the whole population had grade 0 comorbidities, for the purpose of our analysis, we divided patients into two comorbidity categories: grade 2 or grade <2.

A total of 500 patients from the electronic records of the PD clinic were evaluated; 3 were excluded due to administrative issues, leaving 497 for analysis.

### Outcomes

The study evaluated the following outcomes: the transition from PD to HD, the likelihood of transplantation and the all-cause mortality rate. We defined the failure of the technique as the definitive transition from PD to HD. Temporary switches from PD to HD for clinical reasons (e.g. peritonitis, catheter malfunctions or abdominal surgery) lasting less than 30 days were not considered as causes of technique failure. The determinants of transition were categorized into several factors, which included inadequate dialysis, catheter-related mechanical complications, peritonitis, psychosocial causes and abdominal surgeries.

### Statistical analysis

To conduct the statistical analysis, we employed the R software, version 4.3.3. In our presentation of data, we relied on measures of central tendency and dispersion, expressing quantitative variables as means ± standard deviation and qualitative variables as frequencies. To facilitate statistical inference, we employed the χ^2^ test and Student's *t*-test according to the types of variables. We utilized the Kaplan–Meier (KM) method to assess both technique and patient survival, employing the log-rank test for the comparison of survival curves [2].

To account for competing risks and improve the accuracy of survival estimates, we implemented Fine and Gray's subdistribution hazard model to analyze patient and technique survival, treating death, transition to HD and transplantation as competing events. Additionally, we use the cause-specific Cox proportional hazards model [2] to provide complementary insights into independent risk factors influencing each outcome separately. The rationale for using both models lies in their complementary strengths. Fine and Gray's model estimates the subdistribution hazard and is useful for clinical decision-making, while the cause-specific Cox model provides insight into the biological risk of each event.

Censoring was applied as follows: in patients survival analysis, censoring was applied to individuals lost to follow-up, those who underwent kidney transplantation, or those who transitioned to HD. In technique survival analysis censoring was applied for patients lost to follow-up, those who passed away or those who received a kidney transplant.

We estimate the cumulative incidence function (CIF) and compared with 1-KM estimates. Subgroup analyses using Gray's test [3], were conducted to explore differences based on patient characteristics.

To assess whether the division into study periods introduced interaction effects, we tested an interaction between the study period and key covariates in both the Fine and Gray and cause-specific Cox models. No significant interaction was found, justifying the use of time period as an independent covariate in the analysis.

For variables selection in the multivariable models, an exploratory analysis was conducted by fitting univariable models and selecting covariates with *P* < .10 for inclusion in the multivariable models.

The mean percentage of missing data was 24%. To account for missing data in the regression models, we applied multiple imputation for chained equations (MICE) using predictive mean matching (mice R package, version 3.9.0). Given the mean percentage of missing data, the number of imputations for all missing values was set to 24. To ensure convergence, we performed 20 iterations for each imputation.

## RESULTS

### Patient characteristics

The study enrolled 497 PD patients. Main demographic characteristics are summarized in Table 1. The mean age at the start of PD was 63.5 ± 15.7 years. The majority of the patients were males (61% of the cohort), 61% had three or more comorbidities including hypertension (81%), dyslipidemia (66%), cardiovascular disease (56%), hyperuricemia (49%) and DM (16%). Only 25% of the patients were treated with RAASi. The mean eGFR at the initiation of PD was 7.3 ± 1.9 mL/min/1.73 m^2^, and continuous ambulatory peritoneal dialysis (CAPD) was the preferred modality for 49% of patients.

**Table 1:** Study population.

Characteristic	*N* = 497
Age (years)	63.5 (15.7)
Gender (male), *n* (%)	303 (61)
DM, *n* (%)	78 (16)
eGFR at the start of dialysis, mL/min	7.3 ± 1.9
Hypertension, *n* (%)	404 (81)
CAPD, *n* (%)	246 (49)
RAASi use, *n* (%)	126 (25)
Hypercholesterolemia *n* (%)	327 (66)
Hyperuricemia, *n* (%)	246 (49)
Cardiovascular disease, *n* (%)	279 (56)
Comorbidities (>3), *n* (%)	301 (61)

Data are presented as mean (standard deviation), or n (%).

Data on the causes of ESKD were available in 427 out of 497 patients. The main causes of ESKD are reported in Table 2. Table 3 summarizes differences among the three activity periods considered.

**Table 2:** Main causes of ESKD.

Causes	*n*/*N* (%)
Glomerular disease (primary or secondary)	109/427 (25.5)
Hypertension	84/427 (19.7)
DM	76/427 (17.8)
Tubulointerstitial disease	39/427 (9.1)
Cystic kidney diseases	32/427 (7.5)
Vascular disease	31/427 (7.3)
Urinary tract obstruction or dysfunction	4/427 (0.9)
Drugs-induced	10 (2.3)
Other known causes	10 (2.3)
Unknown causes	32 (7.5)

**Table 3:** Study population according to activity period.

		Study period^a^			
Characteristic	*N* = 497	Group 1, *n* = 119 (23.9)	Group 2, *n* = 186 (37.4)	Group 3, *n* = 192 (38.6)	*P*-value
Age (years), mean ± SD (range)	63.5 (15.7)	63.6 (17.3)	64.1 (15.3)	63.2 (15.1)	.8
Gender (female), *n* (%)	194 (39.0)	61 (51)	60 (32)	73 (38)	.004
DM, *n* (%)	78 (16)	0	22 (12)	56 (29)	<.001
eGFR at the start of dialysis, mL/min, mean ± SD	7.3 ± 1.9	7.6 (1.6)	7.2 (1.9)	7.2 (2.0)	.053
Hypertension, *n* (%)	404 (81)	80 (67)	151 (81)	173 (90)	<.001
CAPD, *n* (%)	246 (49)	65 (55)	90 (48)	91 (47)	.4
RAASi use, *n* (%)	126 (25)	26 (22)	59 (32)	41 (21)	.041
Hypercholesterolemia *n* (%)	327 (66)	75 (63)	109 (59)	143 (74)	.004
Hyperuricemia, *n* (%)	246 (49)	37 (31)	84 (45)	125 (65)	<.001
Cardiovascular disease, *n* (%)	279 (56)	46 (39)	130 (70)	103 (54)	<.001
Comorbidities (>3), *n* (%)	301 (61)	34 (29)	116 (62)	151 (79)	<.001
Catheter type, *n* (%)					<.001
Vicenza single-cuff catheter	58 (12)	57 (48)	0 (0)	1 (0.5)	
Vicenza double-cuff catheter	16 (3.2)	14 (12)	0 (0)	2 (1.0)	
Self-locating catheter	41 (8.2)	16 (13)	7 (3.8)	18 (9.4)	
Swan neck straight catheter	326 (66)	32 (27)	126 (68)	168 (88)	
Swan neck coiled catheter	56 (11)	0 (0)	53 (28)	3 (1.6)	
Outcome					<.001
Alive in PD	64 (12.9)	1 (0.8)	0 (0.0)	63 (32.8)	
Transition to HD	204 (41.0)	52 (43.7)	88 (47.3)	64 (33.3)	
Kidney transplant	92 (18.5)	23 (19.3)	33 (17.7)	36 (18.7)	
Died	137 (27.6)	43 (36.1)	65 (34.9)	29 (15.1)	

^a^Study period Group 1 (1996–2005), Group 2 (2006–15) and Group 3 (2016–21).

### Reasons for discontinuing peritoneal dialysis

The mean follow-up was 2.9 ± 2.4 years. A total of 204 patients (41%) transitioned from PD to HD, 137 patients (27.5%) died, 92 (18.5%) received kidney transplant and 64 (13%) remained alive on PD.

The patients who received a transplant were significantly younger (49.8 ± 12 years), had a higher prevalence of hypertension (93%) and a lower prevalence of DM (3.3%). In this group, automated peritoneal dialysis (APD) was the preferred modality for 79%.

The patients who transitioned from PD to HD received CAPD in 52% of cases. The primary cause of PD failure was attributed to inadequate dialysis (34.3% of cases). Another notable cause was peritonitis, accounting for 23% of cases. Mechanical complications accounted for 7.8%, and cases involving abdominal surgeries represented 6.9%. Psychosocial factors, such as patient or caregiver burnout, were identified as contributing to 8.3% of PD failures. The causes of PD failure remained unknown in 16.2% of cases.

Multivariable model analysis for PD-to-HD transfer are summarized in Table 4.

**Table 4:** Multivariable models for transition from PD to HD.

	Cox model		Subdistribution model	
Covariates	HR (95% CI)	*P*-value	SHR (95% CI)	*P*-value
Gender (female)	0.70 (0.51–0.96)	.028	0.93 (0.69–1.26)	.700
Age >75 years	1.02 (0.71–1.45)	>.9	0.70 (0.49–1.02)	.062
eGFR at the beginning of PD	0.92 (0.85–1.00)	.051	0.88 (0.82–0.95)	<.001
DM	0.96 (0.63–1.45)	.800	1.28 (0.85–1.93)	.200
CAPD	1.62 (1.21–2.16)	.001	1.15 (0.85–1.54)	.400
Hypertension	1.33 (0.89–1.99)	.200	1.36 (0.93–1.99)	.110
Hyperuricemia	1.20 (0.89–1.63)	.200	1.13 (0.83–1.54)	.500
RAASi	0.66 (0.46–0.94)	.021	0.72 (0.50–1.02)	.063
More than 3 comorbidities	1.13 (0.78–1.63)	.500	0.81 (0.56–1.17)	.300
Activity period				
Group 1	Ref.		Ref.	
Group 2	1.34 (0.92–1.97)	.130	1.08 (0.76–1.55)	.700
Group 3	1.91 (1.22–2.99)	.005	1.02 (0.67–1.56)	>.9

SHR, subdistribution HR.

**Figure 1:**
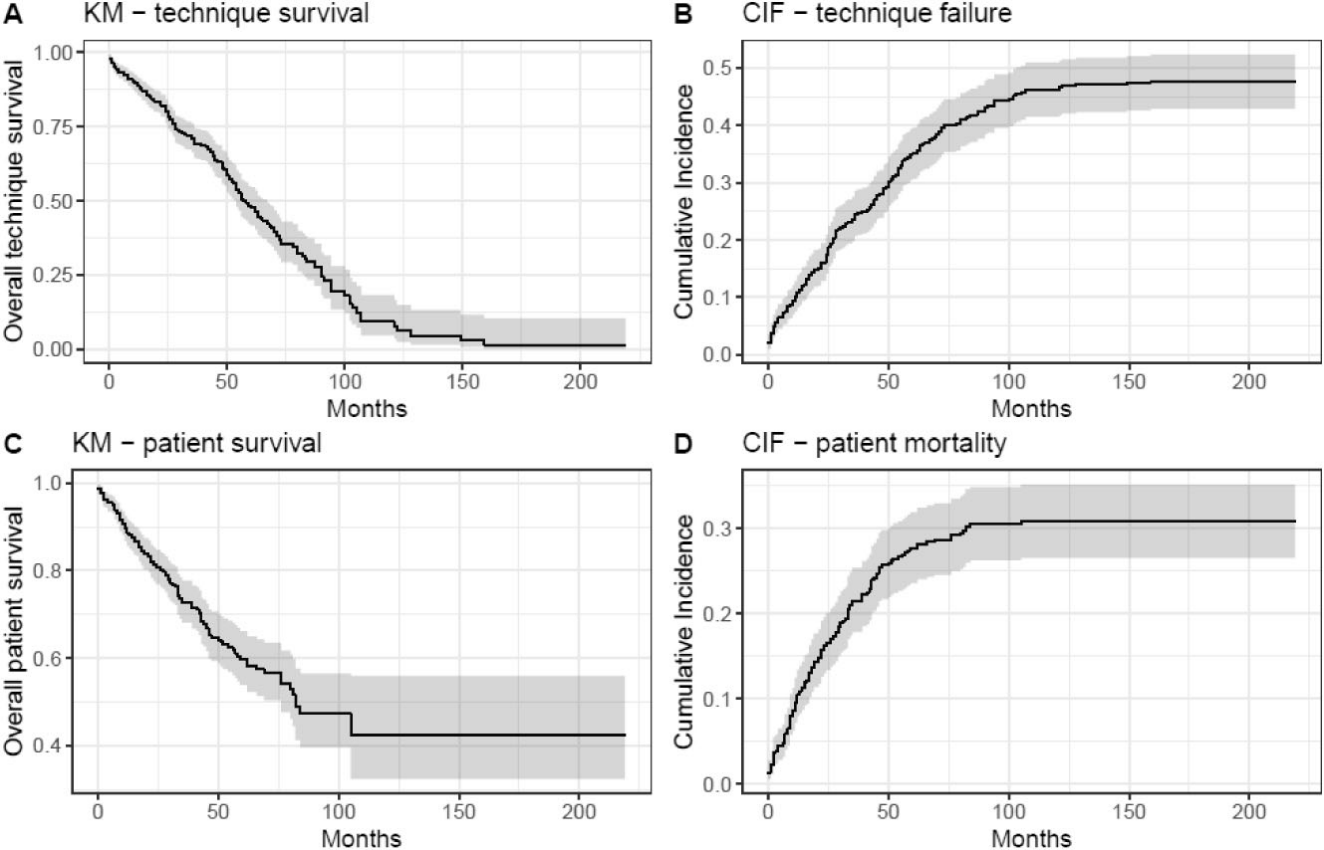
KM curves for (**A**) technique survival and (**C**) patient survival. CIF curves for (**B**) technique failure and (**D**) patient mortality.

In the analysis of PD-to-HD transfer using Cox proportional hazards model, the persistence on PD rate was 88%, 69%, 48% and 30% at 1-, 3-, 5- and 7-year intervals, respectively (Fig. 1). The median time on PD therapy was 57 [95% confidence interval (CI) 54–67] months. Risk factors for transition from PD to HD included CAPD use [hazard ratio (HR) = 1.62, 95% CI 1.21–2.16; *P* = .001]. The use of RAASi and female gender were associated with a better longer time on PD therapy: the HR were 0.66 (95% CI 0.46–0.94; *P* = .02) and 0.70 (95% CI 0.51–0.96; *P* = .03), respectively. Furthermore, male patients anticipated the transition to HD by 1.1 years compared with their female counterparts (*P* = .006).

The cumulative incidence of transition from PD to HD was 11%, 25%, 35% and 42% at 1-, 3-, 5- and 7-year intervals, respectively (Fig. 1). Using Gray subdistribution hazard model, eGFR at the beginning of PD was associated with an HR of 0.88 (95% CI 0.82–0.95; *P* < .001).

### Analysis of patient survival

The all-cause mortality rate among PD patients was 100.3 per 1000 person-years. For patients aged over 75 years, the mortality rate reached 242.1 per 1000 person-years.

Multivariable model analysis for patient survival are summarized in Table 5.

**Table 5:** Multivariable models for patient survival.

		Cox model	Subdistribution model	
Covariates	HR (95% CI)	*P*-value	SHR (95% CI)	*P*-value
Hyperuricemia			0.79 (0.53–1.17)	.200
DM			1.60 (0.92–2.79)	.094
Gender (female)	0.73 (0.52–1.05)	.087		
CAPD	3.05 (2.07–4.51)	<.001	2.63 (1.76–3.93)	<.001
Hypertension	0.77 (0.53–1.12)	.200	0.63 (0.41–0.96)	.034
Hypercolesterolemia	0.60 (0.43–0.86)	.005	0.72 (0.49–1.04)	.080
Cardiovascular disease	1.99 (1.32–2.98)	<.001	1.96 (1.31–2.95)	.001
Age >75 years	3.05 (2.13–4.35)	<.001	2.96 (1.98–4.43)	<.001
RAASi use			1.81 (1.22–2.70)	.004
Activity period				
Group 1			Ref.	
Group 2			0.72 (0.40–1.29)	.300
Group 3			0.44 (0.22–0.86)	.017
Catheter type				
Vicenza single-cuff catheter			Ref.	
Vicenza double-cuff catheter			1.77 (0.85, 3.67)	.12
Self-locating catheter			4.39 (2.28, 8.47)	<.001
Swan neck straight catheter			1.26 (0.61, 2.62)	.500
Swan neck coiled catheter			2.84 (1.11, 7.27)	.029

SHR, subdistribution HR.

In the analysis of survival using Cox proportional hazards model, the 1-, 3-, 5- and 7-year survival rates were 89%, 73%, 60% and 47%, respectively (Fig. 1). Multivariate Cox regression analysis revealed age >75 years (HR = 3.05, 95% CI 2.13–4.35; *P* < .001), CAPD use (HR = 3.05, 95% CI 2.07–4.51; *P* < .001) and cardiovascular disease (HR = 1.99, 95% CI 1.32–2.98; *P* < .001) as significant risk factors. Hypercholesterolemia was associated with an HR of 0.60 (95% CI 0.43–0.86; *P* = .005).

The cumulative incidence of mortality was 10%, 22%, 28% and 30% at 1-, 3-, 5- and 7-year intervals, respectively (Fig. 1). Risk factors for mortality included CAPD use (HR = 2.63, 95% CI 1.76–3.93; *P* < .001), age >75 years (HR = 2.96, 95% CI 1.98–4.43; *P* < .001), the use of RAASi (HR = 1.81, 95% CI 1.22–2.70; *P* = .004) and the presence of cardiovascular disease (HR = 1.96, 95% CI 1.31–2.95; *P* = .001). Notably, patients with hypertension had an HR of 0.63 (95% CI 0.41–0.96; *P* = .034).

### Comparisons among different activity periods

Comparing demographics among the three study periods, there were no differences in mean age at the time of dialysis start. Patients from the first decade were generally more balanced in gender distribution whether Group 2 and 3 showed a higher prevalence of males. Group 1 patients had a lower prevalence of comorbidities. Notably, only 29% of patients had three or more comorbidities (*P* < .001) and the whole Group 1 did not include diabetic patients.

**Figure 2:**
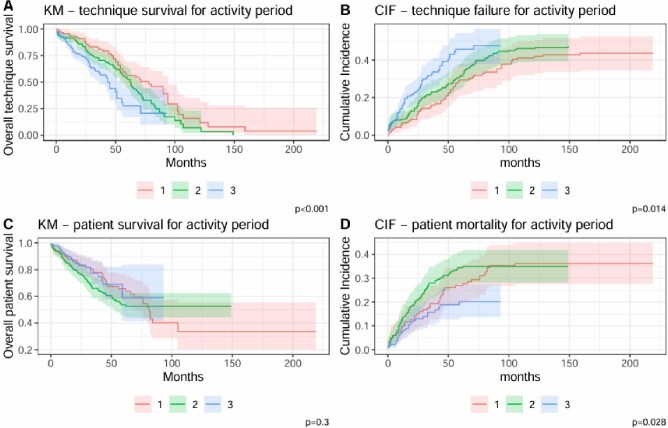
KM curves for (**A**) technique survival and (**C**) patient survival. CIF curves for (**B**) technique failure and (**D**) patient mortality. Curves are stratified by activity periods: (1) 1996–2005, (2) 2006–15, (3) 2016–21.

Over the three study periods, we observed a significant increase in PD discontinuation without any significant change in the percentage of patients receiving kidney transplant (18%–19%), or any significant improvement in patient survival rates on Cox proportional hazards model (Fig. 2).

While distribution by activity period was not identified as risk factor on Cox model, using Gray subdistribution hazard model for assessing mortality, Group 3 was associated with an HR of 0.44 (95% CI 0.22–0.86; *P* = .017).

In the analysis of technique survival using either Gray subdistribution hazard model or Cox proportional hazards model, distribution by activity period was not identified as risk factor, although KM showed better results for Group 1 (*P* < .001) (Fig. 2).

Using Gray's test, calculating 5-year cumulative incidence for transition from PD to HD according to the three activity periods (Fig. 2) revealed a higher cumulative incidence for the more recent period [46% vs 29% (Group 1) and 32% (Group 2), *P* = .014]. Subgroup analyses, using Gray's test, performed calculating 5-year cumulative incidence for mortality according to the three timeframes revealed a lower cumulative incidence for the more recent period [20% vs 27% (Group 1) and 34% (Group 2), *P* = .02].

## DISCUSSION

Patient demographics in our cohort are similar to other Italian and European cohorts [7, 8]. In the 8th National Census of Peritoneal Dialysis in Italy, the main causes of ESKD in PD patients were nephroangiosclerosis (24.4%), glomerulonephritis (22.3%) and diabetic nephropathy (15.4%) [7]. Our study cohort exhibited a 16% prevalence of diabetic patients. Primary kidney disease etiologies predominantly included DM and glomerular diseases (Table 2). Moreover, the demographic characteristics of our PD patients have changed significantly over time, closely mirroring national trends [7]. We have observed a higher prevalence of females compared with males, a substantial increase in diabetic patients, reaching up to 29%, along with a general increase in comorbidities, especially a rise in the incidence of hypercholesterolemia and hyperuricemia. Notably, despite the high prevalence of comorbidities in our cohort, only a quarter of patients received therapy with RAASi. Given the well-documented benefits of RAASi in the PD population, this limited adoption is concerning.

It is noteworthy that, no diabetic patients were enrolled in PD during the first decade of activity (1996–2005). Certainly, this finding may be partially explained by the inherent limitations of a retrospective study, where the accurate retrieval of comorbidities could be affected by incomplete or missing data. However, another key factor to consider is the clinical decision-making process at the time.

During the late 1990s and early 2000s, growing skepticism regarding PD in diabetic patients was fueled by the fear of potential complications, including a presumed higher risk of peritonitis, ultrafiltration failure, and adverse metabolic and nutritional consequences [9]. Although some of these concerns were based on evidence, others might have been influenced by prevailing misconceptions rather than solid data [9]. This historical context may have influenced the clinical choices made in our center during that period, leading to the absence of diabetic patients in our PD program. Interestingly, the later introduction of glucose-sparing dialysis solutions, such as icodextrin and amino acid–based solutions, helped extend the benefits of PD to diabetic patients by mitigating metabolic effects.

### Patient survival

Our cohort all-cause mortality rate was 100.3 per 1000 person-years. The Netherlands Cooperative Study on the Adequacy of Dialysis (NECOSAD) trial [8] observed a 2-year patient survival in incident PD patients of 77%, a percentage not different from our overall 3-year survival of 73%. In the 8th Italian PD census the number of deaths recorded in 2022 was 400 corresponding to a mortality rate of 100.1 per 1000 person-years [7].

Our study revealed an overall 5-year patient mortality rate of 40%, with age emerging as the leading factor influencing patient survival. Interestingly, CAPD use was also identified as a risk factor. We can only speculate on the clinical significance of this observation.

One possible interpretation relates to peritoneal membrane transport characteristics. High peritoneal solute transport characteristics, especially in patients on CAPD, early in the course of treatment has been associated with both reduced patient survival and technical failure [10–12]. This is thought to result from faster glucose absorption, leading to diminished ultrafiltration, fluid overload and potential cardiovascular complications [10, 11]. Unfortunately, we do not have data on the distribution of patients according to Peritoneal Equilibration Test in our cohort.

Alternatively, patient selection bias may better explain the association between CAPD and adverse outcomes. In our cohort, CAPD patients were significantly older than those on APD (68.3 ± 15 vs 59.2 ± 15, *P* < .001), whereas no difference in comorbidities was seen. However, we acknowledge that our study did not use a validated comorbidity index, which may have limited our ability to detect subtle differences in baseline health status.

Moreover, the observation that the kidney transplant rate was significantly higher in APD patients compared with CAPD patients (27% vs 9.2%, *P* < .001) further suggests that patients treated with APD had a more favorable overall health profile, which could have contributed to the observed differences in outcomes.

Thus, although CAPD was associated with a higher mortality risk, this finding likely reflects differences in patient characteristics rather than an inherent limitation of the modality.

Another factor related to the dialysis technique that showed an effect on mortality in our analysis was the type of catheter used. The observed higher HR for mortality associated with self-locating and swan neck coiled catheters may reflect a historical shift in patient characteristics rather than a true causal effect of the catheters. The reference group, consisting of patients treated in an earlier era, had a more favorable clinical profile, which could partly explain the differences in survival outcomes.

Another unexpected finding was the association of hypertension with an HR of 0.63. Foley *et al*. [13] have previously shown a relationship between mean arterial blood pressure and cardiovascular morbidity, but they could not establish an association between mean arterial blood pressure and death. Nevertheless, data from other studies have proven a clear relationship between hypertension and death [13–16]. Notably, Zager *et al*. [17] have demonstrated the presence of a “U” curve relationship between systolic blood pressure post-dialysis and cardiovascular mortality in HD patients, nonetheless neither systolic nor diastolic hypertension, measured pre-dialysis, was associated with an increase of cardiovascular mortality; whether such findings are generalizable to PD patients remains unknown. An alternative explanation can be found in the retrospective nature of our study and the criteria used to define hypertension. Since hypertension in our study was only inferred by the use of antihypertensive drugs, we were unable to assess the type and degree of hypertension as well as the presence of untreated hypertension among those defined as non-hypertensive patients at the baseline. Furthermore, during the study period, the therapeutic thresholds for the treatment of hypertension have repeatedly and deeply changed [18].

Similar considerations could also apply to hypercholesterolemia in Cox proportional hazards model, which showed an HR of 0.60. On the whole, this finding is not surprising since reverse epidemiology of mortality risk factors is a well-known phenomenon when comparing maintenance dialysis patients to the general population [19].

The association of RAASi use with an increased risk of mortality also deserves some comments. This finding may be related to the dose of drug which is normally never prescribed at top values in dialysis patients due to the risk of hyperkalemia. Given the retrospective nature of our study, we could not quantify the prevalence of hyperkalemia and its relationship with mortality risk in this cohort. However, patients on PD are generally more prone to hypokalemia than hyperkalemia and RAASi are generally considered safe and useful in PD patients [20, 21].

### Technique survival

The 3-year technique survival in our series was 73%, slightly higher than that observed in the NECOSAD trial (64% at 2 years) [8]. The primary cause of PD failure was attributed to inadequate dialysis in 34.3% of cases. Another notable factor contributing to the shift from PD to HD was peritonitis, accounting for 23% of cases. Despite the causes of PD failure remained unknown in 16.2% of cases, these percentages are not dissimilar to those reported from the 8th National Census of Peritoneal Dialysis in Italy which reported inadequate dialysis (including both ultrafiltration failure and inadequate clearance) as the leading cause (26.7%) and peritonitis as the second most common cause (23.5%) [7].

In our study, CAPD was associated with an increased technique failure, probably for the same reason responsible for the association with mortality. The better survival of PD technique in patients on RAASi therapy and with a higher eGFR at the start of dialysis is a common observation. Several studies have shown potential beneficial effects of RAASi on prevention of peritoneal fibrosis and preservation of residual kidney function [20].

Therefore, in our study, the use of RAASi, such as ramipril, presented a nuanced impact on both patient mortality and technique survival. One possible explanation for this dual effect involves the dosing strategies commonly employed in clinical practice.

For effective cardiovascular benefits, higher doses of ramipril are generally needed [20–22]. However, to mitigate the risk of hyperkalemia, clinicians might prescribe ramipril at lower doses, which have been shown to reduce proteinuria without significantly affecting blood pressure or increasing plasma potassium levels [21, 23].

On the other hand, even at these lower doses, ramipril has been shown to provide benefits for technique survival in peritoneal dialysis. For example, a dose of 5 mg daily has been demonstrated to slow the decline in residual renal function [24]. This suggests that while the dose might be suboptimal for cardioprotection, it may still be sufficient to confer some degree of nephroprotection and contribute to the longevity of the dialysis technique. Alternatively, the use of RAASi might serve as a marker for patients with existing cardiovascular comorbidities, who inherently have a higher risk of mortality. However, due to the retrospective nature of our study, we were unable to verify this hypothesis.

### Technological advances and temporal trends in PD outcomes

In our cohort, stratification by activity periods allowed us to assess the clinical impact of key innovations in PD management [25, 26]. The transition to biocompatible dialysis solutions occurred between the first and second periods, while remote telemonitoring was introduced in the third. These advances are considered pivotal in enhancing patient outcomes: biocompatible solutions have been shown to preserve residual renal function and urine output [25]. Telemonitoring has been associated with improved survival and fewer adverse events and hospitalizations [26].

Consistent with these observations, we found a lower cumulative incidence of death in the most recent period, despite a higher prevalence of comorbidities and DM due to a wider uptake of PD. Conversely, technique survival did not follow a similar trend. The 5-year cumulative incidence of PD discontinuation was significantly higher in the most recent period compared with earlier ones (46% vs 29% and 32%; *P* = .014). This likely reflects both the increasing complexity of the treated population and earlier recognition of complications enabled by telemonitoring, which may have prompted timely transitions to HD.

Overall, while technological advancements have enhanced clinical management and patient survival, they do not appear to have fundamentally altered the intrinsic technique survival of PD as a treatment modality. In this context, changes in patient selection and management may have influenced technique outcomes more than innovations in PD itself.

## CONCLUSIONS

While our study provides valuable insights on the changing landscape of PD, certain inherent limitations must be acknowledged. As a single-center retrospective study, our study findings might not be generalizable to all settings, since center-specific factors may have influenced survival outcomes. Our study did not assess patient comorbidity using a robust index as a metric [5] We did not comprehensively address key variables, such as serum albumin, residual renal function and peritonitis rates, which could impact patient or technique survival. Additionally, although temporal trends were considered, we did not specifically assess the direct contribution of individual innovations such as biocompatible solutions or remote telemonitoring to the observed outcomes. Based on these observations, a multicenter study involving a larger patient cohort could help validate our findings.

## Data Availability

The data underlying this article will be shared on reasonable request to the corresponding author.
